# Delayed Migration of a Transcatheter Aortic Valve Into the Left Ventricular Outflow Tract

**DOI:** 10.1016/j.jaccas.2025.104437

**Published:** 2025-07-30

**Authors:** Arsh Issany, Dany F. Debs, Lana Abusubaih, Mohamed Abdel-Ahmed, Vijay Iyer

**Affiliations:** a Jacobs School of Medicine and Biomedical Sciences, Buffalo, New York, USA; b Roger Williams Medical Center, Providence, Rhode Island, USA; c Buffalo General Hospital/Gates Vascular Institute, Buffalo, New York, USA

**Keywords:** aortic valve, echocardiography, valve replacement

## Abstract

**Background:**

Late device migration is a rare complication of the transcatheter aortic valve replacement procedure.

**Case Summary:**

This is a 60-year-old man with a medical history of surgical aortic valve replacement with subsequent transcatheter aortic valve replacement who presented to the emergency department for chest discomfort and dyspnea. The transthoracic echocardiogram showed moderate prosthetic aortic regurgitation and severe prosthetic aortic valve stenosis, with evidence of valve migration. The patient underwent a successful transcatheter aortic valve-in-valve replacement.

**Discussion:**

Transcatheter heart valve migration, typically occurring shortly after deployment, is uncommon in late presentations, with few cases reported. Migration occurs when retrograde forces overcome the strength of the valve's endothelial attachment. Previous surgical aortic valve replacement, worsening heart failure, and endothelial dysfunction may have played a role in migration.

**Take-Home Messages:**

Transcatheter heart valve migration is a potentially lethal complication. Patients presenting with acute heart failure and known bioprosthesis require prompt evaluation with echocardiography.

## Graphical Abstract

**Figure:**
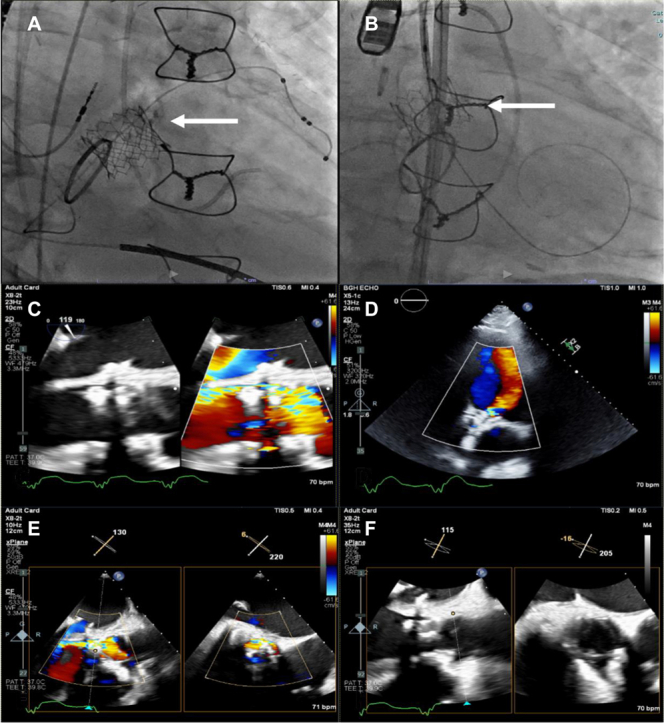

Transcatheter aortic valve replacement (TAVR) has revolutionized how we treat aortic stenosis. TAVR has similar short- and long-term all-cause mortality in moderate- and high-risk patients as surgical aortic valve replacement (SAVR).^1^ Avoiding surgery has allowed TAVR to be a gold standard for high-risk patients.^2^ However, there are some complications. Device migration is a rare and life-threatening complication of the TAVR procedure, with a periprocedural rate of 0.9%.^3^

**Take-Home Messages**

• Transcatheter heart valve migration can be a lethal complication of the procedure.
• Patients presenting with acute heart failure with known bioprosthesis should be quickly evaluated with echocardiography.
• Patients with high surgical risk can undergo the valve-in-valve procedure.

## History of Presentation

A 60-year-old man with a past medical history significant for SAVR (25-mm Medtronic Freestyle aortic root heart valve for aortic insufficiency [AI]) in 2014, mitral valve repair (30-mm Edwards Physio annuloplasty ring for mitral regurgitation) in 2014, subsequent TAVR in 2016 (26-mm Edwards Sapien pericardial tissue heart valve for acute AI secondary to a torn leaflet of aortic valve prostheses), ischemic heart failure with reduced ejection fraction (HFrEF) with a left ventricular ejection fraction (LVEF) of 20% to 25%, status post biventricular implantable cardioverter-defibrillator, deep vein thrombosis, and pulmonary embolism presented to the emergency department for a 2-day history of chest discomfort and dyspnea.

## Past Medical History

The patient had an additional history of coronary artery disease status post stenting (mid-left anterior descending artery) in 2005, coronary artery bypass grafting × 2 (saphenous vein graft from the aorta to the right posterior ascending vein and left internal mammary artery graft from the aorta to diagonal branch 2) in 2014, ischemic HFrEF with an LVEF of 20% to 25% with a biventricular implantable cardioverter-defibrillator, and deep vein thrombosis.

## Differential Diagnosis

Because of the patient's history of HFrEF and prior TAVR in SAVR, differential diagnoses included HFrEF exacerbation, myocardial infarction, and transcatheter heart valve (THV) dysfunction.

The patient's known bioprosthesis, new onset dyspnea, and signs of volume overload prompted evaluation of the bioprosthesis with a transthoracic echocardiogram (TTE)/transesophageal echocardiogram (TEE), which indicated THV migration.

## Investigations

**Figure 1:**
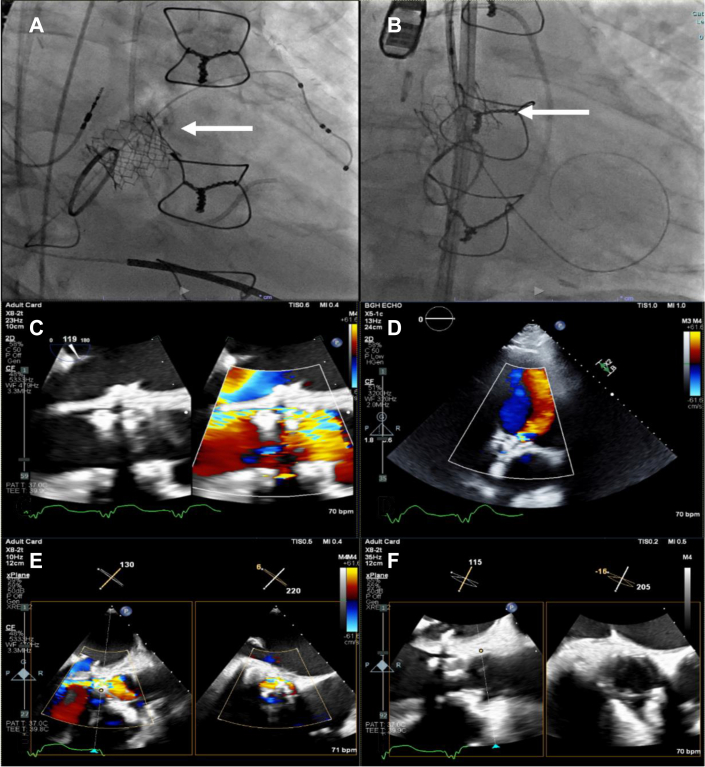
Imaging Demonstrating TAVR Valve Migration Migration of the TAVR valve into the LVOT (A); note the relative position of the TAVR valve with the previous SAVR (arrow). TAVR valve good position before migration (B). TEE at 119° showing severe paravalvular leak and migration of the TAVR valve down into the LVOT (C). TTE 4-chamber view showing severe paravalvular regurgitation in red (D). TEE at 130° X plane showing severe paravalvular regurgitation (E). TEE at 115° X plane showing the TAVR valve migrated into the LVOT (F). Note the short axis image at the aortic valve annulus level showing the SAVR valve leaflets without the TAVR valve present (indicating an incorrect position). LVOT = left ventricular outflow tract; SAVR = surgical aortic valve replacement; TAVR = transcatheter aortic valve replacement; TEE = transesophageal echocardiogram.

TTE, performed to assess the THV, showed moderate central aortic prosthetic regurgitation, severe aortic prosthetic stenosis, and LVEF 20% to 25% (Figure 1). TEE subsequently showed migration of the THV into the left ventricular outflow tract (LVOT), and TAVR leaflets thickened and restricted with severe stenosis (Figure 1). Because of high surgical risk, a transcatheter aortic valve-in-valve replacement was pursued.

## Management

A 26-mm Edwards Lifesciences SAPIEN 3 Ultra Resilia bioprosthetic valve (nominal + 3 cc) was placed in the aortic position with rapid ventricular pacing. The final position of the valve was appropriate as there was no residual aortic stenosis, and no significant aortic regurgitation was noted. A second inflation was performed (at nominal +3 cc) in an attempt to expand the prior Edwards valve from 2016 that had migrated into the left ventricle. TEE confirmed that bioprosthesis was well seated with no abnormal rocking motion. Leaflet morphology and mobility appeared adequate. The previously noted protruding native leaflets were mostly pinned behind the newly deployed THV. No residual gradient, with a peak of 25 mm Hg (249 cm/s) and a mean of 10 mm Hg, was noted. The estimated aortic valve area was 1.6 cm^2^. No significant central aortic regurgitation or paravalvular leak was observed.

## Outcome and Follow-Up

The next day, TTE showed good prosthetic valve position, expansion, and normal leaflet mobility. No significant residual stenosis with normal gradients and no significant central or paravalvular regurgitation were noted. The patient was discharged the following day after reporting significantly improved symptoms. He followed up in the clinic a couple of weeks later and denied any new symptoms.

## Discussion

Delayed THV migration is a rare and feared complication of the procedure. The most common periprocedural complications are moderate/severe paravalvular leak (PVL), major vascular and bleeding complications, stroke, acute kidney injury, and atrioventricular block requiring a pacemaker.^4^ Only a few cases have been reported in the literature, the longest one being 4 years after the procedure and the rest occurring a few weeks after. This case presented to us 8 years after the procedure, making it the longest reported so far.^3^^,^^5^, ^6^, ^7^

Currently, it is hypothesized that valvular migration occurs when retrograde forces overcome the strength of the THV's attachment to the endothelium.^8^ Migration can occur perioperatively because of low implantation, anatomical changes (bicuspid valve), and calcifications.^6^^,^^9^ Long-term migration has not been studied extensively because it is a much rarer complication. Factors that cause migration perioperatively can also affect long-term migration as well as changes in the patient's condition. There were a few factors that may have predisposed this patient to valve migration.

The patient had a significant cardiac history with a prior SAVR. This was replaced with TAVR in 2016 where the patient presented with chest pain and dyspnea. TEE before TAVR in 2016 showed prolapse or torn leaflet of the initial aortic valve prostheses. Post-TAVR TEE in 2016 showed that bioprosthetic valve is well seated with no significant perivalvular regurgitation. The leaflets are well seen and have normal motion. Peak/mean gradients and aortic valve area were 14/6 mm Hg and 1.7 cm^2^, respectively. There was no central aortic regurgitation. We have also attached the initial TAVR placement in 2016, which shows proper positioning (Video 1).

The patient's cardiac history may have predisposed the subsequent THV to fail. The torn/prolapsed valve may have altered the fit and disrupted the endothelialization process. Smoking is known to cause endothelial dysfunction.^10^ This may have halted the endothelialization process that would have strengthened the adherence of the valve to the aortic wall. Another reason for long-term migration is poor anchoring. The initial TAVR valve in 2016 was deployed in a freestyle valve without calcium. This may have decreased the anchoring force. The patient also had a left ventricular (LV) cavity diastolic diameter of 50 mm in 2016, 54 mm in 2018 without significant AI/PVL, 56 mm in 2019 without significant AI/PVL, and 66 mm 1 day after TAVR echocardiogram in 2024. The progressive LV dilation over time is likely not due to AI/PVL as the serial TTEs ruled that out; rather it is due to progression of the underlying heart failure. This increasing LV cavity size could have altered the geometry of the LVOT and aortic root and increased the likelihood of migration.

## Conclusions

This is a case of a very delayed valve migration after TAVR in SAVR. Many factors could have contributed to this, such as unfavorable anatomy causing decreased adherence to the endothelium, increased retrograde pressure or altered LVOT geometry due to LV dilation. The delayed presentation of our patient points to a nonoperative etiology. This further stresses the importance of long-term follow-up and recognition of symptoms.

**Visual Summary:** Timeline of Key Events

Timeline	Events
SAVR in 2014	Patient presented with symptomatic heart failure and received SAVR for aortic insufficiency.
TAVR in SAVR in 2016	Patient again presented with symptomatic heart failure. TEE showed a torn SAVR leaflet. Patient received TAVR in SAVR.
Initial presentation in 2023	Patient presented with a 2-d history of chest discomfort and dyspnea. TEE showed migration of the THV into the LVOT. Patient undergoes the valve-in-valve procedure.
1-d postoperatively	Patient reported improved symptoms. TTE showed good prosthetic valve position.
1-mo postoperatively	Patient presented to the outpatient clinic and denied any new symptoms.

SAVR = surgical aortic valve replacement; TAVR = transcatheter aortic valve replacement; TEE = transesophageal echocardiogram.

## Funding Support and Author Disclosures

The authors have reported that they have no relationships relevant to the contents of this paper to disclose.

## Appendix

Video 1 Initial Post-TAVR Aortic Root Angiogram From 2016
